# D-glucose Stimulates the Na^+^/K^+^ Pump in Mouse Pancreatic Islet Cells

**DOI:** 10.1155/EDR.2000.155

**Published:** 2000

**Authors:** Adrian Elmi, Lars-ÅKe Idahl, Per-Erik Sandström, Janove Sehlin

**Affiliations:** 1 Department of Integrative Medical Biology Section for Histology and Cell Biology Umed University Umed SE-901 87 Sweden

## Abstract

To determine the effect of D-glucose on the β-cell Na^+^/K^+^ pump, ^86^Rb^+^ influx was studied in isolated, -cell-rich islets of Umeå-*ob/ob* mice in the absence or presence of lmM ouabain. D-glucose (20 mM) stimulated the ouabain-sensitive portion of ^86^Rb^+^ influx by 65%, whereas the ouabain-resistant portion was inhibited by 48%. The Na^+^/K^+^ ATPase activity in homogenates of islets of Umeå-*ob/ob* mice or normal mice was determined to search for direct effects of D-glucose. Thus, ouabain-sensitive ATP hydrolysis in islet homogenates was measured in the presence of different D-glucose concentrations. No effect of D-glucose (3–20 mM) was observed in either *ob/ob* or normal islets at the optimal Na^+^/K^+^ ratio for the enzyme (135 mM Na^+^ and 20 mM K^+^). Neither D-glucose (3–20 mM) nor L-glucose or 3-*O*-methyl-D-glucose (20 mM) affected the enzyme activity at a high Na^+^/K^+^ ratio (175 mM Na^+^ and 0.7mM K^+^). Diphenylhydantoin (150 μM) decreased the enzyme activity at optimal Na^+^/K^+^ ratio, whereas 50 μM of the drug had no effect. The results suggest that D-glucose induces a net stimulation the Na^+^/K^+^ pump of β-cells in intact islets and that D-glucose does not exert any direct effect on the Na^+^/K^+^ ATPase activity.


*Int. Jnl. Experimental Diab. Res.*, Vol. 1, pp. 155–164
Reprints available directly from the publisher
Photocopying permitted by license only

© 2000 OPA (Overseas Publishers Association) N.V.
Published by license under
the Harwood Academic Publishers imprint,
part of The Gordon and Breach Publishing Group.
Printed in Malaysia.

### D-glucose Stimulates the $Na^+/K^+$ Pump in Mouse Pancreatic Islet Cells

ADRIAN ELMI, LARS-ÅKE IDAHL, PER-ERIK SANDSTRÖM and JANOVE SEHLIN*

*Department of Integrative Medical Biology, Section for Histology and Cell Biology, Umeå University, SE-901 87 Umeå, Sweden*

*(Received 30 December 1999; In final form 12 February 2000)*

**To determine the effect of D-glucose on the β-cell $Na^+/K^+$ pump, $^{86}Rb^+$ influx was studied in isolated, -cell-rich islets of Umeå-*ob/ob* mice in the absence or presence of 1 mM ouabain. D-glucose (20 mM) stimulated the ouabain-sensitive portion of $^{86}Rb^+$ influx by 65%, whereas the ouabain-resistant portion was inhibited by 48%. The $Na^+/K^+$ ATPase activity in homogenates of islets of Umeå-*ob/ob* mice or normal mice was determined to search for direct effects of D-glucose. Thus, ouabain-sensitive ATP hydrolysis in islet homogenates was measured in the presence of different D-glucose concentrations. No effect of D-glucose (3–20 mM) was observed in either *ob/ob* or normal islets at the optimal $Na^+/K^+$ ratio for the enzyme (135 mM $Na^+$ and 20 mM $K^+$). Neither D-glucose (3–20 mM) nor L-glucose or 3-*O*-methyl-D-glucose (20 mM) affected the enzyme activity at a high $Na^+/K^+$ ratio (175 mM $Na^+$ and 0.7 mM $K^+$). Diphenylhydantoin (150 μM) decreased the enzyme activity at optimal $Na^+/K^+$ ratio, whereas 50 μM of the drug had no effect. The results suggest that D-glucose induces a net stimulation the $Na^+/K^+$ pump of β-cells in intact islets and that D-glucose does not exert any direct effect on the $Na^+/K^+$ ATPase activity.**

*Keywords*: Insulin, ions, *ob/ob*-mouse, ouabain, $Na^+/K^+$ pump, β-cell

### INTRODUCTION

$Na^+/K^+$ ATPase is important for many aspects of normal cell function. Its precise role in the stimulus–secretion coupling in pancreatic β-cells has been a matter of discussion. In early studies of labelled $Na^+$ and $K^+$ ($Rb^+$) transmembrane transport, it was found that β-cells accumulate $^{86}Rb^+$ [1] and exclude $^{22}Na^+$ [1, 2] in an ouabain-sensitive manner. A much higher concentration of intracellular $Na^+$ was found in pancreatic β-cells, as compared with that found in neurons and muscle cells, which was suggested as an indirect evidence for a low $Na^+/K^+$ ATPase activity in the islets. [3] D-glucose (20 mM) was shown not to affect the islet efflux of labelled $Na^+$ [1] or to increase the fractional outflow rate of $Na^+$. [4] It was shown that diphenylhydantoin (DPH), cAMP, caffeine or $Ca^{2+}$ did not affect the $Na^+/K^+$ ATPase activity in microsomes from mouse pancreatic islets, whereas *p*-chloromercuribenzene-sulfonic acid inhibited the ATPase activity by 84%. [5] Other studies

*Corresponding author. Tel.: +4690-786 51 09, Fax: +4690-786 66 96, e-mail: janove.sehlin@histocel.umu.se performed on the $Na^+/K^+$ ATPase activity in crude membrane preparations of rat pancreatic islets demonstrated that glucose, sulphonylureas, somatostatin and diazoxide were without effect on this ATPase activity. [6] Measurements of the $^{86}Rb^+$ influx indicated that glucose [7] does not inhibit the $Na^+/K^+$ pump in pancreatic $\beta$-cells. Also, such experiments suggested that the inhibition of glucose-stimulated insulin release by DPH [8] is not attributable to activation of a $Na^+/K^+$ pump, indeed $^{86}Rb^+$ influx was inhibited by DPH. [1] Electrophysiological studies have indicated that activation of the $Na^+/K^+$ pump can lead to a marked hyperpolarization of the $\beta$-cell membrane in the presence of 10 mM glucose and that this could be inhibited by ouabain, but no effect of glucose on the electrogenic $Na^+/K^+$ pump could be detected. [9] However, a line of studies have shown that D-glucose inhibited the $Na^+/K^+$ ATPase activity in a dose-related manner and several other agents, known to affect insulin secretion from intact $\beta$-cells, also affected the ATPase activity. [10] These data have been interpreted to suggest that direct inhibitory effects of glucose and several other insulin secretagogues on the $Na^+/K^+$ ATPase could have a direct regulatory influence on the $\beta$-cell stimulus-secretion coupling (see [10]). In a more recent study, an inhibitory effect of D-glucose on islet $Na^+/K^+$ ATPase was confirmed and suggested to be mediated by the activation of a distinct intracellular signalling network. [11] In the present work, we have therefore compared the effects of D-glucose on ouabain-sensitive $^{86}Rb^+$ influx (a marker of the $Na^+/K^+$ pump) in intact pancreatic islets and on the $Na^+/K^+$ ATPase activity in homogenized islets. Intact isolated islets or homogenates from $\beta$-cell-rich pancreatic islets of *ob/ob* mouse or normal mouse were used.

### MATERIALS AND METHODS

### Animals and Isolation of Islets

Non-inbred, 7–8 months old, female *ob/ob* mice (Umeå-*ob/ob*) or 6–8 months old normal, lean littermates were used throughout. *ob/ob* Mice and normal mice were handled in exactly the same way throughout the experiments. Pancreatic islets from Umeå-*ob/ob* mice were used because they contain an exceptionally high proportion of $\beta$-cells ($> 90\%$) [12] and the islet hyperplasia provides enough quantities of islet tissue required for this type of experiments. Although the mice from this breeding stock are metabolically abnormal with mild hyperglycaemia, which may be due to a peripheral insulin resistance and hyperphagia, [13, 14] their islets show normal regulation of insulin secretion *in vitro*. [15–17] The high proportion of $\beta$-cells in these *ob/ob*-mouse islets makes it highly probable that the present data on homogenates and intact isolated islets are representative of this cell type.

All mice were fasted overnight, which in the case of *ob/ob* mice leads to a normalization of their blood sugar. [18] The pancreata were digested with collagenase to isolate individual islets. [19] In the case of normal mice, the digested pancreata were applied on discontinuous density gradients and centrifuged at 6,000 rpm for 16 min, in order to separate the islets from fragments of the exocrine pancreas. The gradient consisted of four successive layers of dextrane 31%, 29%, 25% and 11% (w/v) and the islets were collected from the 11% layer.

The medium used was a Krebs–Ringer medium (KRH) with the following salt composition (mM): NaCl, 130; KCl, 4.7; $KH_2PO_4$, 1.2; $MgSO_4$, 1.2; and $CaCl_2$, 2.56. Bovine serum albumin (BSA) at 10 mg/ml and 3 mM D-glucose were added. The medium was buffered with 20 mM Hepes and NaOH to a final pH of 7.4. In each experiment pancreata from two *ob/ob* mice or 10–12 normal mice were used.

### Measurements of $^{86}Rb^+$ Influx

Pancreatic islets were isolated as described above. Then, batches of five islets were preincubated for 60 min at 37°C in KRH medium containing 3 mM D-glucose. After preincubation, islets were incubated for 5 min at 37°C in the same type of basal medium supplemented with 28 μM $^{86}Rb^+$ and 8 μM [6,6′- $^3H$] sucrose as extracellular marker, essentially as previously described. [1] The D-glucose concentration during incubation with isotopes was 0 or 20 mM. After incubation the islets were freeze-dried (−40°C, 0.1 Pa), weighed on a quartz-fibre balance and their radioactive contents measured in a liquid scintillation spectrometer.

### Islet Homogenisation

The islets isolated as above were washed four times in a homogenisation medium with the following composition (mM): sucrose, 250; KCl, 50; MOPS, 10; and EGTA, 1. The final pH was set to 7.2 with 2 M Trisma-base. Approximately 100 islets from *ob/ob* mice were transferred to a polypropylene micro test tube (Milian Instruments S.A., Geneva, Switzerland) and 60 μl homogenisation medium and a glass bead were added. The islets were then homogenised by vibrating the test tube at a frequency of 1 kHz for 30 s followed by a short centrifugation. The test tubes were then immediately frozen at −20°C. In the case of normal mice, islets from 10–12 mice were pooled and homogenised according to the same procedure as described above.

### Assay of $Na^+/K^+$ ATPase

The method for measuring the activity of $Na^+/K^+$ ATPase was adapted from Jørgensen and Skou. [20] The concentrations of $Na^+$ and $K^+$ were generally chosen to give a maximum rate of ouabain-sensitive ATP hydrolysis (135 mM $Na^+$ and 20 mM $K^+$). Previous studies on the cationic dependence of the $Na^+/K^+$ ATPase of mouse pancreatic islets have shown that the enzyme activity at different $Na^+/K^+$ ratios and the corresponding curve for $Na^+$ and $K^+$ activation of the $\beta$-cell enzyme (Sandström, Klaerke and Sehlin, unpublished data) are very similar to the data previously described for the $Na^+/K^+$ ATPase in the kidney. [21,22] The activity of $Na^+/K^+$ ATPase was measured as the difference in amount of inorganic phosphate released in the presence or absence of ouabain (1 mM). In brief, preincubation of aliquots of the homogenate was performed by mixing 5 parts of islet homogenate prepared as described above, 2 parts of 0.65% sodium deoxycholate (DOC) dissolved in water, 2 parts of 20 mM EDTA in water with pH adjusted to 7.2 with imidazole, and 11 parts of imidazole buffer (25 mM) with pH adjusted to 7.0 using 1 M HCl.

To permeabilize the membranes for measurements of $Na^+/K^+$ ATPase from pancreatic $\beta$-cells, the homogenate was preincubated with 75 μM DOC for 15 to 30 min at room temperature, according to Jørgensen and Skou. [21] Control experiments showed that the ouabain-sensitive ATP hydrolysis was increased by about 4 times by the presence of DOC (data not shown). The increased enzyme activity is a direct result of exposure of latent enzyme sites in the preparation. The exposure to detergent leads to the opening of vesicular structures, resulting in free access of substrate and activators to their respective sites on the membrane. [21] The concentration of DOC in the preparation is so low that it is unlikely that the activation is due to binding of DOC to the membranes as such. [21]

Incubation was carried out in a histidine buffer with the following final composition (mM): $Na^+$, 135; $K^+$, 20; $Mg^{2+}$, 2.9 (the $MgCl_2$ was always kept at the same concentration as the highest ATP concentration used in the experiment); $Cl^-$, 149; histidine, 29; and pH 7.4. After the addition of 50 μl ATP (normally 2.9 mM final concentration), pH 7.4 to 450 μl of histidine buffer, the solution was temperature equilibrated for 10 min at 37°C. Then, 25 μl of the preincubated islet homogenate was added to the reaction mixture. The reaction was allowed to proceed for 10 min at 37°C. The test substances were dissolved in the histidine buffer and all tests were performed both in the absence and presence of ouabain (1 mM) in parallel with relevant blanks. The reaction was terminated by the addition of 1 ml of ice-cold reagent, containing 150 mM L-ascorbic acid and 3.7 mM ammonium heptamolybdate, on an ice-bath. After 10 min, 1.5 ml of reagent containing 150 mM sodium-meta-arsenite, 68 mM sodium citrate and 2% (v/v) acetic acid, was added and the mixture was incubated for 10 min at 37°C. The absorbance of the final solution was then read at 850 nm in a Varian DMS 100 spectrophotometer. Enzyme activity was expressed as mmol inorganic phosphate released (= mmol ATP hydrolysed) per g protein and 10 min. The amount of protein was measured spectrophotometrically as previously described. [23]

### Statistics

Statistical significance was evaluated by using the two-tailed Student's *t*-test. Results are expressed as mean ± S.E.M.

### Chemicals

Amersham Pharmacia Biotech, Uppsala, Sweden provided $^{86}Rb^+$ and [6,6′-$^3$H] sucrose. Sodium deoxycholate (DOC), EDTA titriplex II, sodium free, L-histidine, L(+)-ascorbic acid, sodium meta-arsenite, sodium citrate and ammonium heptamolybdate were obtained from Merck, Darmstadt, Germany. EGTA, MOPS, ouabain, imidazole, $Na_2HPO_4$, dextrane and collagenase, type I, were all from Sigma Chemical Co., St. Louis, MO, U.S.A. and BSA (fraction V) from Miles, Slough, U.K. Hepes and ATP, disodium salt, were from Boeringer, Mannheim, Germany. All other chemicals were of analytical grade.

### RESULTS

### Effect of D-glucose on $^{86}Rb^+$ Influx

To estimate the activity of the $Na^+/K^+$ pump in intact $\beta$-cells, $^{86}Rb^+$ ($K^+$ marker) influx was studied in the absence or presence of 1 mM ouabain. Previous experiments have shown that $^{86}Rb^+$ uptake proceeds linearly for at least 5 min, suggesting that the present data reflect unidirectional influx of the ion, and that maximum effect of ouabain is reached at 1 mM. [24] Figure 1 shows the effects of 20 mM D-glucose on $^{86}Rb^+$ influx. The total influx in the absence of ouabain was significantly reduced (21%, $P < 0.001$; $n = 12$) by D-glucose and the ouabain-resistant influx was reduced by 48% ($P < 0.001$; $n = 12$), which is in line with the well established capacity of D-glucose to inhibit $K^+$ channels. [25, 26] On the other hand, the ouabain-sensitive component of the $^{86}Rb^+$ influx was significantly stimulated (65%, $P < 0.005$; $n = 12$) by 20 mM D-glucose.

### Time-dependence of the $Na^+/K^+$ ATPase Reaction

The linearity of the relationship between the incubation time and the amount of ATP hydrolysed was studied (Fig. 2). The reaction was allowed to proceed for 10 to 60 min and the amount of ATP hydrolysed was measured as described in the methods section. The data indicate a linear time-dependence in the ATPase reaction.

### Concentration-dependent Effect of ATP

In order to investigate the effect of ATP on the enzyme kinetics of $Na^+/K^+$ ATPase from the $\beta$-cell-rich pancreatic islets of *ob/ob* mice (Fig. 3), the effect of ATP concentrations ranging from 0.1 to 5 mM were studied in two separate sets of experiments with an overlap at 2.9 mM ATP ($n = 8$ and $n = 7$ respectively). The data represent the ouabain-sensitive ATPase activity. In control experiments ($n = 2$) it was found that 1 mM ouabain did not affect the phosphate release in homogenates in the absence of added ATP, indicating that the data indeed represent the hydrolysis of added ATP. The final pH was kept

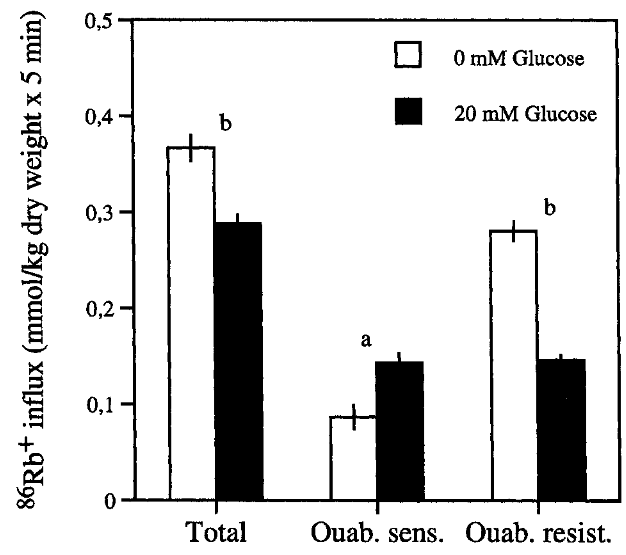

FIGURE 1 Effect of D-glucose on ouabain-sensitive or ouabain-resistant $^{86}Rb^+$ influx. Pancreatic islets were isolated and preincubated as described in the materials and methods and then incubated for 5 min at 37°C in the presence of 28 μM $^{86}Rb^+$ and 8 μM [6,6′- $^3$H] sucrose and in the absence or presence of 20 mM D-glucose and 1 mM ouabain. Bars denote mean values ± SEM for 12 separate experiments. The data are presented as the total influx of $^{86}Rb^+$ at 0 or 20 mM D-glucose ('Total'), the ouabain-sensitive portion of the influx ('Ouab. sens.') and the ouabain-resistant portion of the influx ('Ouab. resist.'). $^aP < 0.005$ and $^bP < 0.001$, for difference between 0 and 20 mM glucose.

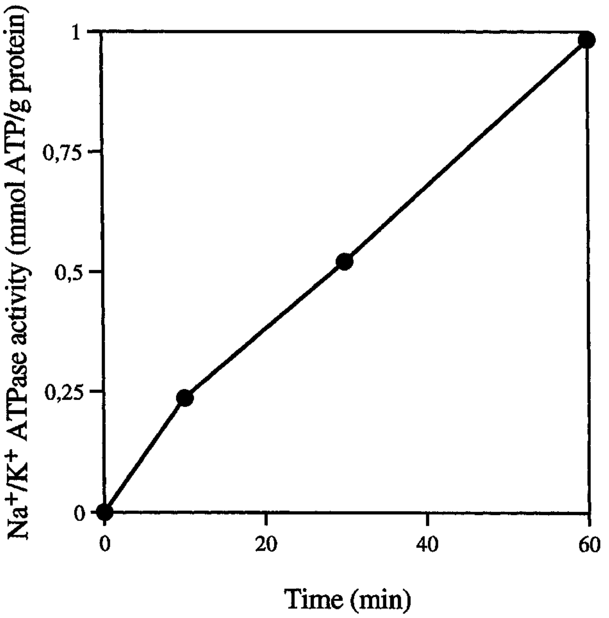

FIGURE 2 Ouabain-sensitive hydrolysis of ATP with time. Homogenates of *ob/ob*-mouse islets were prepared and preincubated as described in the materials and methods and then incubated for 10–60 min at 37°C in one control experiment. The curve has been extrapolated to time zero.

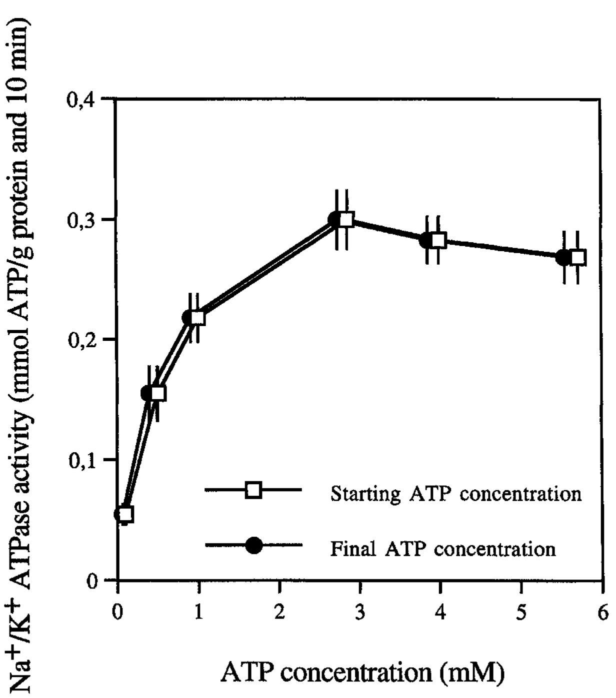

FIGURE 3 Dependence of ouabain-sensitive ATP hydrolysis on the concentration of ATP. Homogenates of *ob/ob*-mouse islets were prepared and preincubated as in Figure 2 and then incubated for 10 min at different ATP concentrations. Symbols (for explanation see inset in figure) denote mean values ± SEM for 7–8 separate experiments.

at 7.4 irrespective of ATP concentration. Figure 3 shows an apparent maximal rate at 3 mM ATP and an apparent $K_{0.5}$ of 0.5 mM ATP. The results are comparable with previous data on $Na^+/K^+$ ATPase in kidney.[22] The rate of ATP consumption during 10 min of incubation was 4% and 21% at 2.9 and 0.5 mM ATP respectively, as calculated from the data in Figure 3.

### Effect of D-glucose on the $Na^+/K^+$ ATPase Activity

As it has been suggested that D-glucose directly affects the $Na^+/K^+$ ATPase of pancreatic islet cells,[10, 11] the effect of D-glucose was studied in islets of both *ob/ob* ($\beta$-cell-rich) and normal mice (Tab. I). The ouabain-sensitive ATP hydrolysis was measured in the presence of 0, 3 or 20 mM D-glucose. In islet homogenates from *ob/ob* mice, no effect of D-glucose was observed (paired or unpaired *t*-test). In experiments with homogenates from normal mice and a D-glucose concentration of 0, 2.6 or 17 mM, also no effect of glucose on ouabain-sensitive ATP hydrolysis was found (paired or unpaired *t*-test).

Since the previously indicated direct inhibitory effects [10] of D-glucose on rat islet $Na^+/K^+$ ATPase activity was demonstrated only at a high ratio of $Na^+$ and $K^+$, we also tested (Tab. II) the effect of D-glucose at this high $Na^+/K^+$ ratio (175 mM $Na^+$ and 0.7 mM $K^+$). The ouabain-sensitive ATP hydrolysis in homogenates from islets of *ob/ob* mice was measured in the presence of 0, 3 or 20 mM D-glucose. Table II shows that also at this high $Na^+/K^+$ ratio, D-glucose did not affect the ouabain-sensitive ATP hydrolysis. Thus, 20 mM D-glucose showed the same lack of effect as L-glucose. Although 3-*O*-methyl-D-glucose showed a slight and probably significant inhibitory effect using paired statistical testing, no effect was evident using unpaired testing and there was no significant

TABLE I Effect of D-glucose on $Na^+/K^+$ ATPase activity at 135 mM $Na^+$ and 20 mM $K^+$

| D-glucose conc. (mM) | ATP conc. (mM) | $Na^+/K^+$ ATPase activity (mmol ATP/g protein and 10 min) |
|---|---|---|
| *ob/ob* Mice | | |
| 0 | 0.5 | 0.228 ± 0.020 (6) |
| 3 | 0.5 | 0.229 ± 0.017 (6) |
| 20 | 0.5 | 0.235 ± 0.016 (6) |
| 0 | 2.9 | 0.277 ± 0.020 (6) |
| 3 | 2.9 | 0.251 ± 0.015 (6) |
| 20 | 2.9 | 0.285 ± 0.020 (6) |
| Normal mice | | |
| 0 | 2.9 | 0.290 ± 0.035 (8) |
| 2.6 | 2.9 | 0.308 + 0.039 (8) |
| 17.1 | 2.9 | 0.329 ± 0.026 (8) |

Homogenates of *ob/ob* or normal mouse islets were prepared and preincubated as described in the materials and methods and then incubated for 10 min at 37°C in the presence of 0.5 ($K_{0.5}$) or 2.9 (maximal rate) mM ATP and different D-glucose concentrations. Data is expressed as mean values ± SEM for the number of separate experiments given in parentheses.

TABLE II Effect of D-glucose, L-glucose or 3-*O*-methyl-D-glucose on $Na^+/K^+$ ATPase activity in islets of *ob/ob* mice at 175 mM $Na^+$ and 0.7 mM $K^+$

| | $Na^+/K^+$ ATPase activity (mmol ATP/g protein and 10 min) | | | |
|---|---|---|---|---|
| | | | Students *t*-test | |
| Sugar concentration (mM) | Primary data | Difference from control | Paired | Unpaired |
| 0 (Control) | 0.107 ± 0.007 (51) | – | | |
| D-glucose, 3 | 0.121 ± 0.011 (14) | 0.017 ± 0.011 | n.s. | n.s. |
| D-glucose, 20 | 0.095 ± 0.006 (51) | −0.012 ± 0.010 | n.s. | n.s. |
| L-glucose, 20 | 0.111 ± 0.008 (26) | 0.021 ± 0.016 | n.s. | n.s. |
| 3-*O*-methyl-D-glucose, 20 | 0.103 ± 0.007 (21) | −0.025 ± 0.011 | $P < 0.05$ | n.s. |

Homogenates of *ob/ob*-mouse islets were prepared and preincubated as described in the materials and methods and then incubated for 10 min at 37°C in the presence of different sugars. Data is expressed as mean values and difference from control ± SEM for the number of experiments indicated in parentheses. n.s. denotes $P > 0.05$ for difference from control. There were no significant differences between the test groups ($P > 0.05$ using either paired or unpaired *t*-test).

TABLE III Effect of DPH on $Na^+/K^+$ ATPase activity in islets of *ob/ob* mice at 135 mM $Na^+$ and 20 mM $K^+$

| | $Na^+/K^+$ ATPase activity (mmol ATP/g protein and 10 min) | | | |
|---|---|---|---|---|
| | | | Students *t*-test | |
| DPH concentration (μM) | Primary data | Difference from control | Paired | Unpaired |
| 0 (Control) | 0.244 ± 0.019 (6) | – | | |
| 50 | 0.256 ± 0.034 (6) | 0.012 ± 0.033 | n.s. | n.s. |
| 150 | 0.179 ± 0.017 (5) | −0.065 ± 0.022 | $P < 0.05$ | $P < 0.05$ |

Homogenates of *ob/ob*-mouse islets were prepared and preincubated as described in the materials and methods and then incubated for 10 min at 37°C in the presence of different concentrations of DPH. Data is expressed as mean values and difference from control ± SEM for the number of experiments indicated in parentheses. n.s. denotes $P > 0.05$ for difference from control.

difference between the test groups. L-glucose is not transported, while 3-*O*-methyl-D-glucose is transported into the $\beta$-cells by the D-glucose transporter but not being metabolised and not inducing insulin secretion (see [27]). The rate of ouabain-sensitive ATP hydrolysis at the high $Na^+/K^+$ ratio was only 42% of the activity at the low ratio.

**Effect of Diphenylhydantoin (DPH)**

Table III shows that DPH at 150 μM caused a statistically significant ($P < 0.05$; $n = 5$) decrease of the ouabain-sensitive ATP hydrolysis by 27%, whereas at 50 μM the drug lacked effect on the enzyme activity.

### DISCUSSION

Previous data from direct or indirect measurements of $Na^+/K^+$ ATPase activity in pancreatic islets are conflicting.[1-11] Direct studies on the $Na^+/K^+$ ATPase activity in microsomes from mouse pancreatic islets showed that it was not affected by DPH, cAMP, caffeine or $Ca^{2+}$, whereas *p*-chloromercuribenzene-sulfonic acid caused an inhibition.[5] Glucose, sulphonylureas, somatostatin and diazoxide had no effect on the $Na^+/K^+$ ATPase activity in crude membrane preparations of rat pancreatic islets.[6] Levin *et al.* [10] found that D-glucose directly inhibited the $Na^+/K^+$ ATPase activity in homogenized rat islets and that several other agents, known to affect insulin secretion from intact $\beta$-cells, also affected the ATPase activity.[10] Recently, an inhibitory effect of glucose on mouse islet cell $Na^+/K^+$ ATPase was observed in experiments using a two-step technique, including incubation of intact islets or isolated cells at different glucose levels and subsequent assay of $Na^+/K^+$ ATPase activity after permeabilization of the islet cells. [11] To test the idea that the $Na^+/K^+$ pump in the pancreatic $\beta$-cells is inhibited by D-glucose, we used the established marker of this pump in intact tissue, the ouabain-sensitive $^{86}Rb^+$ influx (see[1]). The results show that, indeed, the total $^{86}Rb^+$ influx rate was lowered by D-glucose, which is also in agreement with previous findings.[24] When the islet $^{86}Rb^+$ influx was dissected further into ouabain-sensitive and ouabain-resistant portions, it became evident that the glucose-induced inhibition was confined to the ouabain-resistant portion. This observation is in agreement with current knowledge of glucose-induced closure of $K^+$ channels in the $\beta$-cells.[26] However, these experiments also showed that D-glucose increased the ouabain-sensitive fraction of the $^{86}Rb^+$ influx, suggesting that the $Na^+/K^+$ pump in intact $\beta$-cells was accelerated rather than inhibited by the high glucose concentration. Here, it is of interest to recall the previous measurements of $\beta$-cell $Na^+$ content, showing a marked glucose-induced decrease in the $\beta$-cell total content of $Na^+$ [28,29] as well as the cytoplasmic $Na^+$ activity.[29] These observations are in line with the present findings. It is well established that glucose causes a rhythmic pattern of depolarizations and repolarizations of the $\beta$-cell membrane (for review [26]) and a role for an electrogenic $Na^+/K^+$ pump in the repolarization process has been proposed.[9] Our present data thus seem to be in consonance with current ideas on $\beta$-cell membrane potential regulation [9,26] and suggesting that the electrogenic $Na^+/K^+$ pump is stimulated in a secondary manner following the glucose-induced depolarization. However, they do not conform well to the recent data by Owada *et al.* [11], suggesting a pronounced inhibitory effect of glucose on the $Na^+/K^+$ ATPase in intact $\beta$-cells. The reasons for this discrepancy are not easy to define. But it cannot be excluded that differences in methodology are involved. Thus, the conclusions based on the two-step method used by Owada *et al.*[11] seem to require that the islet cells possess a memory function for the $Na^+/K^+$ ATPase activity from the previous treatment period. In order to be able to measure a glucose effect with the experimental design used, this memory has to last for at least 10–25 minutes. However, as also shown by Owada *et al.*,[11] the proposed inhibitory effect of 15 mM glucose on the $Na^+/K^+$ ATPase activity was reversed within 10 min of subsequent exposure to 3 mM glucose, which strongly argues against such a memory function.

The present data on islet $Na^+/K^+$ ATPase show that when the $Na^+/K^+$ ratio and concentrations were chosen to optimise the ouabain-sensitive ATP hydrolysis, D-glucose either at low or high concentrations did not significantly affect the ouabain-sensitive ATP hydrolysis in islet homogenates from either *ob/ob* or normal mice. Also in the presence of the same $Na^+$ and $K^+$ concentrations (175 mM $Na^+$ and 0.7 mM $K^+$) used by Levin *et al.* [10] to show direct effects of glucose on $Na^+/K^+$ ATPase activity, we did not observe any inhibitory effect of glucose. Indeed, there was no difference between D-glucose and L-glucose concerning their lack of effect on $Na^+/K^+$ ATPase activity. The small decrease by 3-*O*-methyl-D-glucose, which is of questionable statistical significance, is difficult to relate to $\beta$-cell secretory regulation in the way previously suggested for D-glucose[10] as 3-*O*-methyl-D-glucose does not stimulate insulin secretion. [27] This finding is likely to represent a chance phenomenon.

That DPH at 150 μM caused an inhibition of ouabain-sensitive ATP hydrolysis at low $Na^+/K^+$ ratio is in consonance with the previous observation that DPH inhibits $^{86}Rb^+$ uptake in intact islet cells incubated in physiological medium.[1] This is also consistent with the data on brain[30] and rat pancreatic islets,[10] showing that DPH at lower $Na^+/K^+$ ratios causes inhibition. A stimulatory effect of DPH on the enzyme activity was found only at high $Na^+/K^+$ ratios.[10,30] The observation of only inhibitory DPH effects on $^{86}Rb^+$ uptake in intact islets under conditions allowing insulin secretion [1,8] indicates that DPH-induced stimulation of $Na^+/K^+$ ATPase does not occur in intact $\beta$-cells and does not explain DPH-induced inhibition of stimulated insulin secretion (*cf.* [8]). It also strongly suggests that the $Na^+$ and $K^+$ concentrations used here, which are associated with DPH inhibition and high enzyme activity, are relevant for physiological function of the enzyme in the intact $\beta$-cell.

In conclusion, the present study indicates that D-glucose, at maximum stimulatory levels for insulin secretion, induces a net stimulatory effect on the $Na^+/K^+$ pump in intact pancreatic islets and that D-glucose does not directly affect the $Na^+/K^+$ ATPase activity in homogenized mouse pancreatic islets.

### *Acknowledgements*

This work was supported in part by the Swedish Medical Research Council (12X-4756), the Swedish Diabetes Association and the Elsa and Folke Sahlberg Fund.

### *References*

[1] Sehlin, J. and Täljedal, I.-B. (1974). Transport of rubidium and sodium in pancreatic islets. *J. Physiol. (Lond)*, **242**, 505–515.

[2] Sehlin, J. and Täljedal, I.-B. (1974). Sodium uptake by microdissected pancreatic islets: effects of ouabain and chloromercuribenzene-*p*-sulphonic acid. *FEBS Lett.*, **39**, 209–213.

[3] Bonting, S. L. (1970). Sodium–potassium activated adenosinetriphosphatase and cation transport. In: *Membrane and Ion Tansport*, edited by Bittar, E. E., **1**, 257–363. London, Wiley-Interscience.

[4] Kawazu, S., Boschero, A. C., Delcroix, C. and Malaisse, W. J. (1978). The stimulus-secretion coupling of glucose-induced insulin release. XXVIII. Effect of glucose on $Na^+$ fluxes in isolated islets. *Pflügers Arch.*, **375**, 197–206.

[5] Formby, B., Capito, K. and Hedeskov, C. J. (1976). ($Na^+$,$K^+$)-activated ATPase in microsomes from mouse pancreatic islets. *Acta Physiol. Scand.*, **96**, 143–144.

[6] Kemmler, W. and Loffler, G. (1977). NaK-ATPase in rat pancreatic islets. *Diabetologia*, **13**, 235–238.

[7] Lebrun, P., Malaisse, W. J. and Herchuelz, A. (1983). $Na^+$–$K^+$ pump activity and the glucose-stimulated $Ca^{2+}$-sensitive $K^+$ permeability in the pancreatic B-cell. *J. Membr. Biol.*, **74**, 67–73.

[8] Herchuelz, A. Lebrun, P., Sener, A. and Malaisse, W. J. (1981). Ionic mechanism of diphenylhydantoin action on glucose-induced insulin release. *Eur. J. Pharmacol.*, **73**, 189–197.

[9] Henquin, J. C. and Meissner, H. P. (1982). The electrogenic sodium–potassium pump of mouse pancreatic B-cells. *J. Physiol. (Lond)*, **332**, 529–552.

[10] Levin, S. R., Kasson, B. G. and Driessen, J. F. (1978). Adenosine triphosphatases of rat pancreatic islets: comparison with those of rat kidney. *J. Clin. Invest.*, **62**, 692–701.

[11] Owada, S., Larsson, O., Arkhammar, P., Katz, A. I., Chibalin, A. V., Berggren, P.-O. and Bertorello, A. M. (1999). Glucose decreases $Na^+$,$K^+$-ATPase activity in pancreatic $\beta$-cells. An effect mediated *via* $Ca^{2+}$-independent phospholipase $A_2$ and protein kinase C-dependent phosphorylation of the $\alpha$-subunit. *J. Biol. Chem.*, **274**, 2000–2008.

[12] Hellman, B. (1965). Studies in obese-hyperglycemic mice. *Ann. N.Y. Acad. Sci.*, **131**, 541–558.

[13] Stauffacher, W. and Renold, A. E. (1969). Effect of insulin *in vivo* on diaphragm and adipose tissue of obese mice. *Am. J. Physiol.*, **216**, 98–105.

[14] Stauffacher, W., Lambert, A. E., Vecchio, D. and Renold, A. E. (1967). Measurements of insulin activities in pancreas and serum of mice with spontaneous ("Obese" and "New Zealand Obese") and induced (Goldthioglucose) obesity and hyperglycemia, with considerations on the pathogenesis of the spontaneous syndrome. *Diabetologia*, **3**, 230–237.

[15] Hahn, H.-J., Hellman, B., Lernmark, Å., Sehlin, J. and Täljedal, I.-B. (1974). The pancreatic $\beta$-cell recognition of insulin secretogogues. Influence of neuraminidase treatment on the release of insulin and the islet content of insulin, sialic acid, and cyclic adenosine 3':5'- monophosphate. *J. Biol. Chem.*, **249**, 5275–5284.

[16] Hellman, B., Idahl, L.-Å., Lernmark, Å., Sehlin, J. and Täljedal, I.-B. (1974). The pancreatic $\beta$-cell recognition of insulin secretagogues. Effects of calcium and sodium on glucose metabolism and insulin release. *Biochem. J.*, **138**, 33–45.

[17] Lindström, P. and Sehlin, J. (1983). Mechanisms underlying the effects of 5-hydroxytryptamine and 5-hydroxytryptophan in pancreatic islets. A proposed role for L-aromatic amino acid decarboxylase. *Endocrinology*, **112**, 1524–1529.

[18] Sandström, P.-E. and Sehlin, J. (1988). Furosemide-induced glucose intolerance in mice is associated with reduced insulin secretion. *Eur. J. Pharmacol.*, **147**, 403–409.

[19] Lernmark, Å. (1974). The preparation of, and studies on, free cell suspensions from mouse pancreatic islets. *Diabetologia*, **10**, 431–438.

[20] Jørgensen, P. L. and Skou, J. C. (1969). Preparation of highly active $(Na^+ + K^+)$-ATPase from the outer medulla of rabbit kidney. *Biochem. Biophys. Res. Commun.*, **37**, 39–46.

[21] Jørgensen, P. L. and Skou, J. C. (1971). Purification and characterization of $(Na^+ + K^+)$-ATPase. I. The influence of detergents on the activity of $(Na^+ + K^+)$-ATPase in preparations from the outer medulla of rabbit kidney. *Biochem. Biophys. Acta*, **233**, 366–380.

[22] Jørgensen, P. L. (1980). Sodium and potassium ion pump in kidney tubules. *Physiol. Rev.*, **60**, 864–917.

[23] Whitaker, J. R. and Granum, P. E. (1980). An absolute method for protein determination based on difference in absorbance at 235 and 280 nm. *Anal. Biochem.*, **109**, 156–159.

[24] Lindström, P., Norlund, L. and Sehlin, J. (1986). Glucose reduces both $Rb^+$ influx and efflux in pancreatic islet cells. *FEBS Lett.*, **200**, 67–70.

[25] Sehlin, J. and Täljedal, I.-B. (1975). Glucose-induced decrease in $Rb^+$ permeability in pancreatic $\beta$-cells. *Nature*, **253**, 635–636.

[26] Ashcroft, F. M. and Rorsman, P. (1989). Electrophysiology of the pancreatic $\beta$-cell. *Prog. Biophys. Mol. Biol.*, **54**, 87–143.

[27] Sehlin, J. (1981). Transport Systems of Islet Cells. In: *The islets of Langerhans: Biochemistry, Physiology and Pathology*, edited by Cooperstein, S. J. and Watkins, D. T., pp. 53–74, New York, Academic Press.

[28] Wesslén, N. and Hellman, B. (1988). Characterization of the glucose-induced lowering of sodium in mouse pancreatic $\beta$-cells. *Acta Physiol. Scand.*, **133**, 11–17.

[29] Ali, L., Grapengiesser, E., Gylfe, E., Hellman, B. and Lund, P.-E. (1989). Free and bound sodium in pancreatic $\beta$-cells exposed to glucose and tolbutamide. *Biochem. Biophys. Res. Commun.*, **164**, 212–218.

[30] Festoff, B. W. and Appel, S. H. (1968). Effect of diphenylhydantoin on synaptosome sodium-potassium-ATPase. *J. Clin. Invest.*, **47**, 2752–2758.

